# Trends in incidence and mortality of esophageal cancer in China 1990−2019: A joinpoint and age-period-cohort analysis

**DOI:** 10.3389/fonc.2022.887011

**Published:** 2022-08-15

**Authors:** Fajun Li, Haifeng Li, Xin Su, Hongsen Liang, Li Wei, Donglei Shi, Junhang Zhang, Zhaojun Wang

**Affiliations:** ^1^ Department of Critical Care Medicine, The First People’s Hospital of Kunshan, Kunshan, China; ^2^ Department of Anesthesiology, Guangdong Provincial People’s Hospital, Guangdong Academy of Medical Sciences, Guangzhou, China; ^3^ Department of Respiratory, Hainan Hospital of PLA General Hospital, Sanya, China; ^4^ Department of Thoracic Surgery, The Seventh Affiliated Hospital, Sun Yat-sen University, Shenzhen, China

**Keywords:** esophageal cancer, age-period-cohort model, joinpoint analysis, incidence trend, mortality trend

## Abstract

**Background:**

The incidence and mortality trends of esophageal cancer (EC) remain unknown in China. This study aimed to describe the trend in incidence and mortality of EC in China.

**Methods:**

We extracted age-standardized rates and numbers of EC in China for 1990−2019 from the Global Burden of Disease study 2019. The age-standardized incidence rate (ASIR) and age-standardized mortality rate (ASMR) were calculated to describe the trends, while the annual percentage of change and the average annual percent change (AAPC) were analyzed by the joinpoint regression analysis. The incidence and mortality data were analyzed *via* age-period-cohort model analysis.

**Results:**

The ASIR and ASMR decreased slightly before 1999, then increased from 1999 to 2004, and decreased again thereafter, with overall AAPC values of −2.5 (−2.8, −2.1) for females and -0.9 (−1.1, −0.8) for males regarding incidence, with overall AAPC values of −3.1 (−3.3, −2.9) for females and −1.2 (−1.3, −1.1) for males regarding mortality. As a whole, the relative risk (RR) of EC increased with age in both females and males regarding incidence and mortality, except for the 80–84-year-old age group in females and the 85–89-year-old age group in males regarding incidence, where they began to decrease. The RR of EC increased with age in females and males regarding mortality, except for the 85–89-year-old age group in males. The time period showed a trend of first rising and then decreasing, and the RR of time period effect was lower in 2015 than that in 1990 in females regarding both incidence and mortality, whereas males showed a significant upward trend in both incidence and mortality. The birth cohort effect showed an overall downward trend.

**Conclusions:**

The overall incidence and mortality of EC in China shows an increased and then decreased trend from 1990 to 2019. The AAPC decreased in incidence and mortality from 1990 to 2019. The RR of incidence and mortality of EC in China is greatly affected by age in both sexes, by time period in male, we should be paid more attention to.

## Introduction

The burden of cancer incidence and mortality is rapidly growing worldwide (1). Esophageal cancer is one of the leading causes of cancer death in the world (2, 3). As a whole, esophageal cancer (EC) ranked seventh in incidence (604,000 new cases) and sixth in mortality (544,000 deaths) in 2020 (3). China still has a heavy cancer burden as the largest developing country in the world (4). Although it may be due to economic growth and improved diets, the incidence of EC in China is in decline (3); however, overall trends may mask differences or veil underlying causes. In order to identify the causes of annual trends in incidence and mortality, it is necessary to examine the annual percent change.

An age-period-cohort model (APCM) is a popular analytical method in both sociological and epidemiological research (5, 6), which can enhance our understanding of incidence and mortality trends by disentangling age, time period, and birth-cohort effects (7), and has been used to analyze the quality and character of cancer prevalence trends. Li et al. used APCM analysis to observe time trends of esophageal and gastric cancer mortality in China from 1991 to 2009 (8). Li et al. explored the trends and risk factors of incidence and mortality in China during 2005-2015 (9).

In this study, we present results from the Global Burden of Disease (GBD) 2019 and provide an assessment of current trends in incidence and mortality of EC in China from 1990 to 2019 by using a joinpoint and age-period-cohort analysis. We hope that our findings can provide a reference for policy planning and contribute to improving cancer control measures in China.

## Methods

### Data sources

The esophageal cancer death number, incidence rate, mortality rate, and national population were obtained from the GBD 2019 study. The GBD study offers a comparative assessment of health loss caused by 328 diseases in 195 countries within 21 regions. Data on the EC were obtained from the Global Health Data Exchange, including individuals aged 20 to 94 years old, from 1990 to 2019, in China (http://ghdx.healthdata.org/gbd-results-tool). When the data were collected, double check was used. The 10th version International Classification of Diseases

codes C15-C15.9, Z85.01 were mapped to EC cancer from GBD 2019 study. Because the data of EC patients under the age of 20 years old was zero, we excluded this part of the data. The APCM analysis requires five-year intervals for each age group (10), and we excluded groups 95 years old and older.

### Descriptive study

The age-standardized incidence rate (ASIR) and age-standardized mortality rate (ASMR) were adopted to evaluate the trends in China between 1990 and 2019. They were calculated according to a direct method based on the GBD 2019 China age-standardized population.

### Trends analysis

Joinpoint regression was used to analyze trends in the age-standardized EC burden. The joinpoint regression program describes trends by connecting several different line segments at ‘joinpoints’ and identifying points where the linear slope of a trend changes in a statistically significant way over time (11). The slope of each line segment was expressed as annual percent change (APC) and average annual percent change (AAPC) with a best-fitting model (12). The APC represents the incidence and mortality rate of the change per year at different times, and the AAPC was a weighted average of the APCs, with the weights equal to the length of the joinpoint segment (11, 13). In this study, we used the APC and AAPC to describe the annual change in EC incidence and mortality rates from 1990 to 2019. Joinpoint regression software developed by the National Cancer Institute (version 4.1.0) was used.

### Age-period-cohort model analysis

The APCM is developed to reflect cancer incidence and mortality relative risks by estimating the age, time period, and birth cohort effects (14). Because of the period = age + cohort relationship, in this study, the rates of EC incidence and mortality were recoded into successive age groups (20–24, 25–29, 30–34, 35–39, 40–44, 45–49, 50–54, 55–59, 60–64, 65–69, 70–74, 75–79, 80–84, 85–89, 90–94), consecutive 5-year periods (1990, 1995, 2000, 2005, 2010, and 2015) and 20 birth cohorts (1900-1904, 1905-1909, 1910-1914, 1915-1919, 1920-1924, 1925-1929, 1930-1934, 1935-1939, 1940-1944, 1945-1949, 1950-1954, 1955-1959, 1960-1964, 1965-1969, 1970-1974, 1975-1979, 1980-1984, 1985-1989, 1990-1994, and 1995-1999). The APCM intrinsic estimator method presents estimated coefficients for the age, time period, and birth cohort effects, and these coefficients were used to calculate the RR (relative risk (RR) = exp (coef.)) (5). A value of p < 0.05 was considered statistically significant.

For data processing, Stata version 17.0 (StataCorp, College Station, TX, USA) was used. Bayesian information criterion, Akaike information criterion, and deviance were used to estimate the degree of model fit. R software was used for drawing (version 4.1.2).

## Results

### Descriptive analysis of incidence and mortality trends

**Figure 1:**
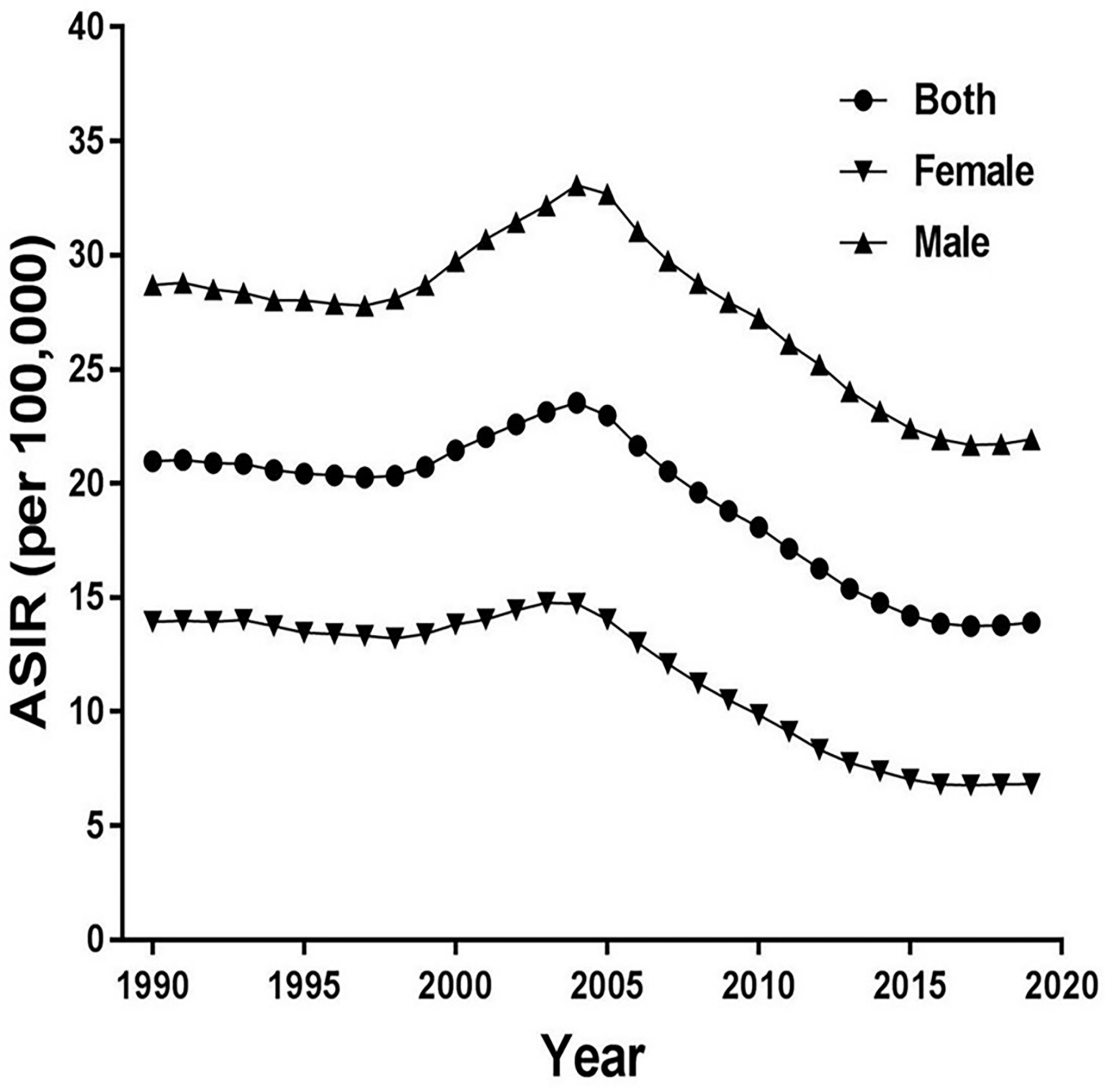
Trends of the age-standardized incidence rates (ASIR) for esophageal cancer from 1990 to 2019 in China.

**Figure 2:**
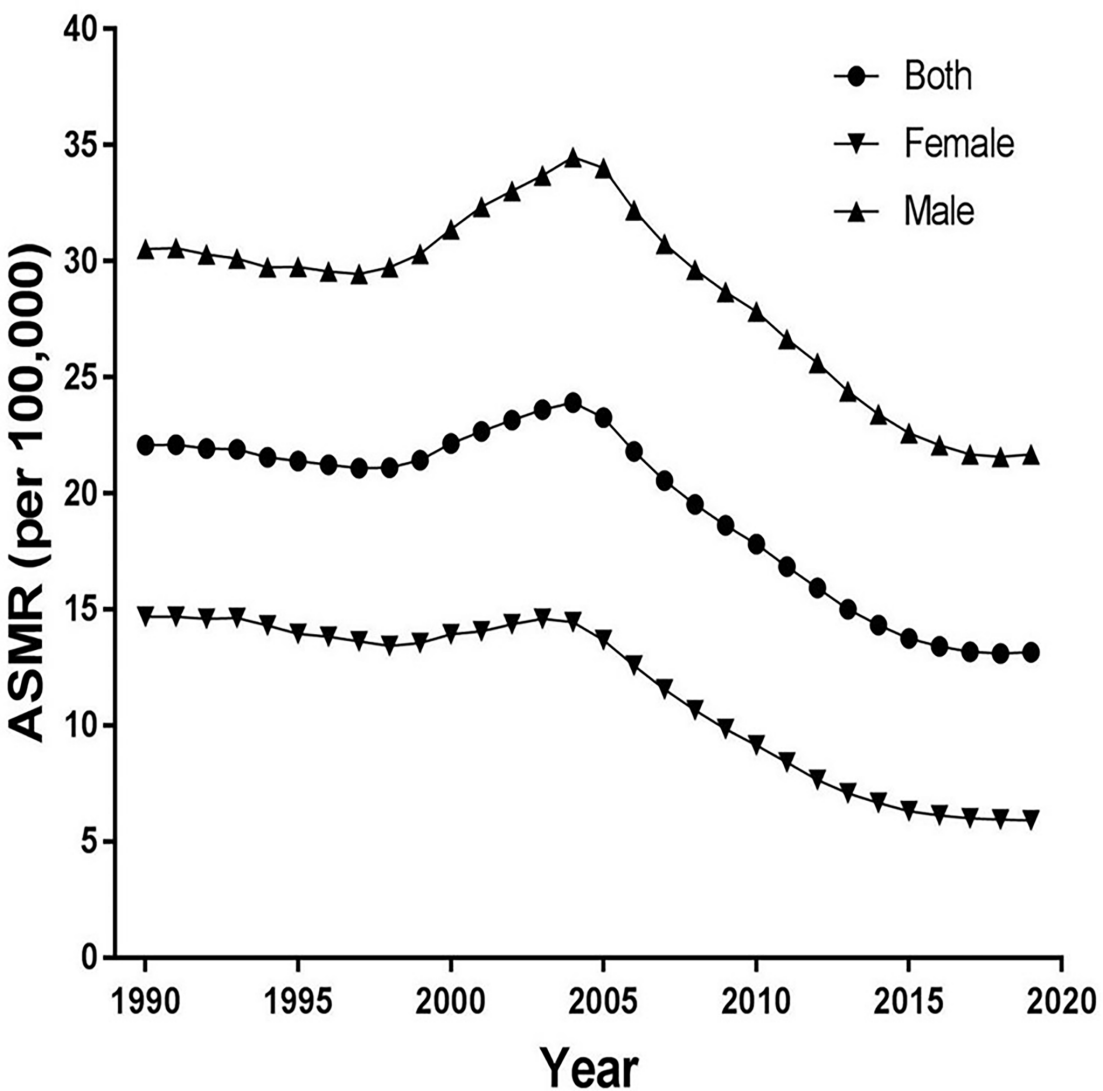
Trends of the age-standardized mortality rates (ASMR) for esophageal cancer from 1990 to 2019 in China.

**Figure 1** shows trends of the ASIR for EC from 1990 to 2019 in China. As a whole, ASIR decreased slightly before 1999, then it gradually rose, peaking in 2004. Obviously, the increase was more pronounced in males, and then decreased markedly. The ASIR decreased from 20.97 in 1990 to 13.90 in 2019 in both sexes, from 13.94 in 1990 to 6.83 in 2019 in females, and from 28.70 in 1990 to 21.94 in 2019 in males per 100,000 persons. In **Figure 2**, the ASMR shows the same curve as in **Figure 1**; the ASMR decreased from 22.08 in 1990 to 13.15 in 2019 in both sexes, from 14.69 in 1990 to 5.92 in 2019 in females, and from 30.53 in 1990 to 21.69 in 2019 in males per 100,000 persons.

**Figure 3:**
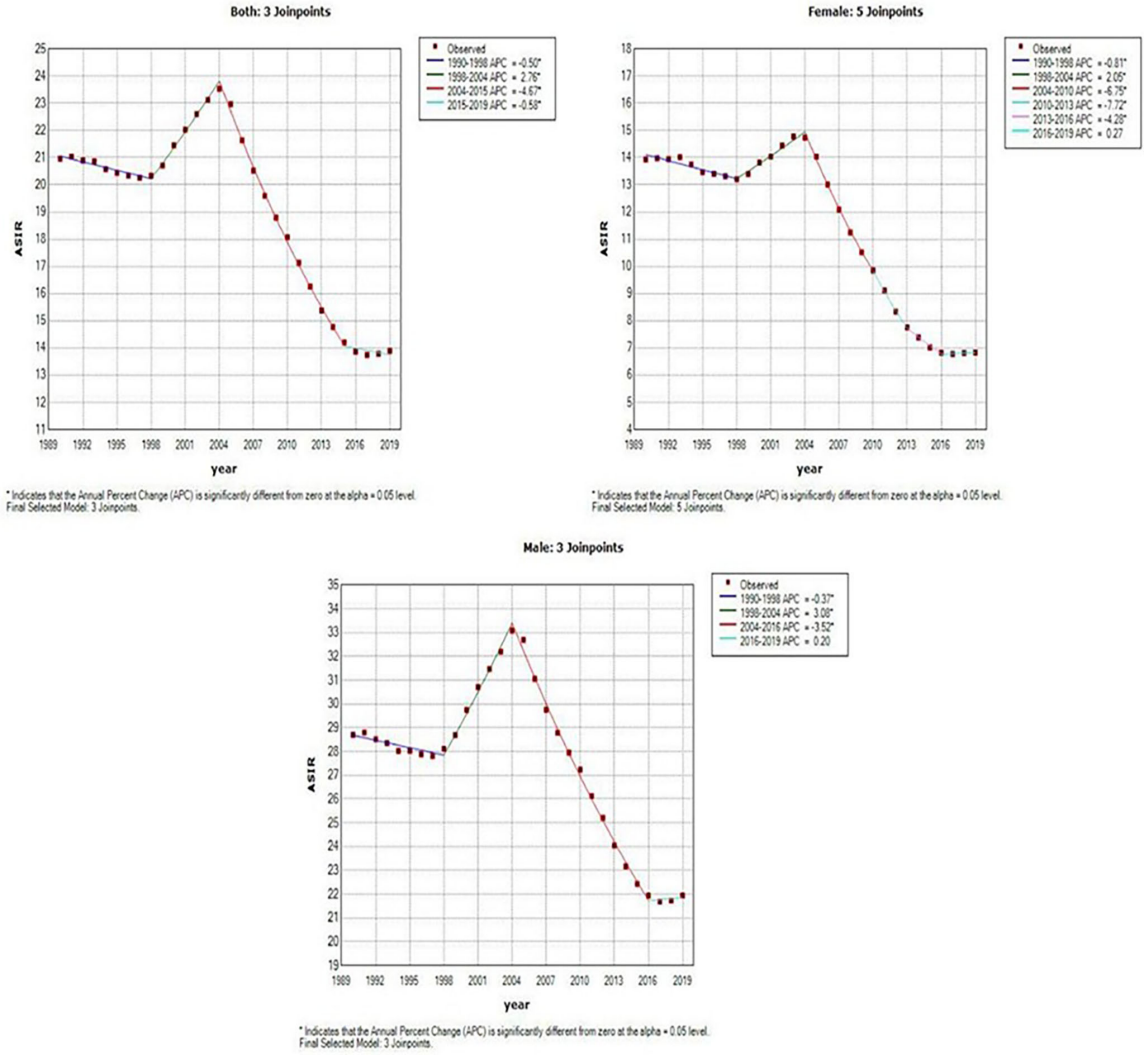
Joinpoint analysis of trends in the age-standardized incidence rates (ASIR) of Esophageal cancer.

**Figure 3** and **Table 1** present the jointpoint analysis of trends in the ASIR of EC in China from 1990 to 2019. The joinpoint regression results show that the ASIR decreased from 1990 to 1998, rose from 1998 to 2004, and decreased again from 2004 to 2019 in both sexes, with overall AAPC values of −1.5 (−1.6, −1.3). In females, the ASIR decreased from 1990 to 1998, rose from 1998 to 2004, decreased from 2004 to 2016, and then rose again from 2016 to 2019. There were six trends in all, with overall AAPC values of −2.5 (−2.8, −2.1). In males, the ASIR had a similar trend as in females over time, but there were four trends in all, with overall AAPC values of −0.9 (−1.1, −0.8).

**Table 1:** Joinpoint analysis of trends in age-standardized incidence rates (ASIR) of esophageal cancer in China, 1990–2019.

Segments	Both			Female			Male		
	Year	APC (95% CI)	AAPC (95% CI)	Year	APC (95% CI)	AAPC (95% CI)	Year	APC (95% CI)	AAPC (95% CI)
Trend 1	1990 - 1998	-0.5* (-0.7,-0.3)	–	1990 - 1998	-0.8* (-1,-0.6)	–	1990 - 1998	-0.4** (-0.6,-0.2)	–
Trend 2	1998 - 2004	2.8* (2.4,3.1)	–	1998 - 2004	2.1* (1.6,2.5)	–	1998 - 2004	3.1* (2.7,3.5)	–
Trend 3	2004 - 2015	-4.7* (-4.8,-4.6)	–	2004 - 2010	-6.7* (-7.2,-6.3)	–	2004 - 2016	-3.5* (-3.6,-3.4)	–
Trend 4	2015 - 2019	-0.6** (-1.1,-0.1)	–	2010 - 2013	-7.7* (-9.7,-5.7)	–	2016 - 2019	0.2 (-0.7,1.1)	–
Trend 5	–	–	–	2013 - 2016	-4.3** (-6.3,-2.2)	–	–	–	–
Trend 6	–	–	–	2016 - 2019	0.3 (-0.8,1.4)	–	–	–	–
AAPC	1990 - 2019	–	-1.5* (-1.6,-1.3)	1990 - 2019	–	-2.5* (-2.8,-2.1)	1990 - 2019	–	-0.9* (-1.1,-0.8)

APC, the annual percentage of change; AAPC, the average annual percent change; CI, confidence interval; ^*^p<0.001, ^**^p<0.05.

**Figure 4:**
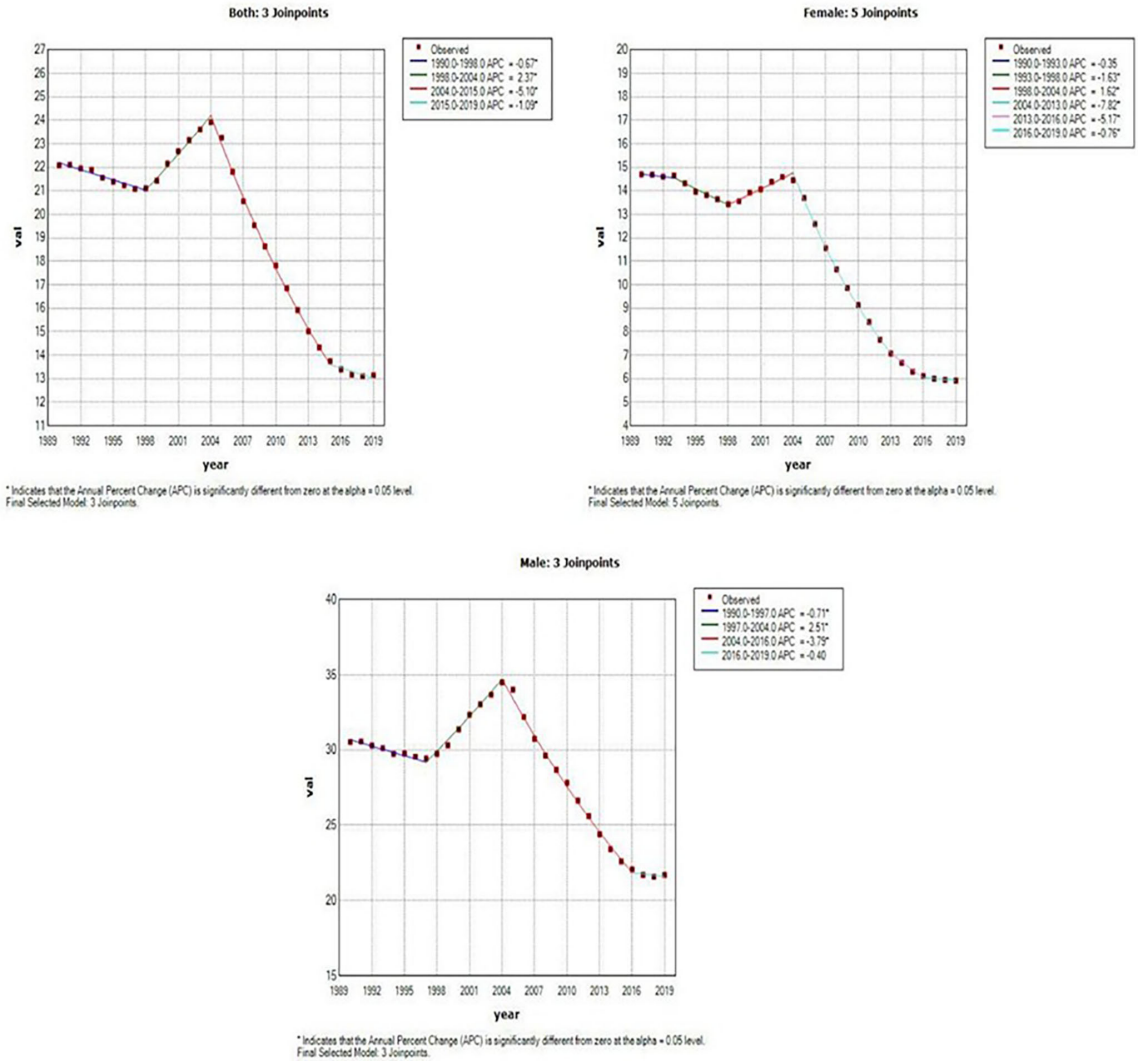
Joinpoint analysis of trends in the age-standardized mortality rates (ASMR) of Esophageal cancer.

**Figure 4** and **Table 2** present the joinpoint analysis of trends in the ASMR of EC in China from 1990 to 2019. The ASMR decreased from 1990 to 1998, rose from 1998 to 2004, and then decreased again from 2004 to 2019 in both sexes, with overall AAPC values of −1.8 (−1.9, −1.7). In females, the ASMR decreased from 1990 to 1998, rose from 1998 to 2004, and decreased again from 2004 to 2019. There were six trends in all, with overall AAPC values of −3.1 (−3.3, −2.9). In males, the ASMR decreased from 1990 to 1997, rose from 1997 to 2004, and then decreased again from 2004 to 2019, with overall AAPC values of -−1.2 (−1.3, −1.1).

**Table 2:** Joinpoint analysis of trends in age-standardized mortality rates (ASMR) of esophageal cancer in China, 1990–2019.

Segments	Both			Female			Male		
	Year	APC (95% CI)	AAPC (95% CI)	Year	APC (95% CI)	AAPC (95% CI)	Year	APC (95% CI)	AAPC (95% CI)
Trend 1	1990 - 1998	-0.7* (-0.8,-0.5)	–	1990 - 1993	-0.4 (-1.1,0.4)	–	1990 - 1997	-0.7* (-0.9,-0.5)	–
Trend 2	1998 - 2004	2.4* (2,2.7)	–	1993 - 1998	-1.6* (-2.1,-1.2)	–	1997 - 2004	2.5* (2.2,2.8)	–
Trend 3	2004 - 2015	-5.1* (-5.2,-5)	–	1998 - 2004	1.6* (1.3,1.9)	–	2004 - 2016	-3.8* (-3.9,-3.7)	–
Trend 4	2015 - 2019	-1.1* (-1.6,-0.6)	–	2004 - 2013	-7.8* (-8,-7.7)	–	2016 - 2019	-0.4 (-1.3,0.5)	–
Trend 5	–	–	–	2013 - 2016	-5.2* (-6.5,-3.8)	–	–	–	–
Trend 6	–	–	–	2016 - 2019	-0.8** (-1.5,-0.1)	–	–	–	–
AAPC	1990 - 2019	–	-1.8* (-1.9,-1.7)	1990 - 2019	–	-3.1* (-3.3,-2.9)	1990 - 2019	–	-1.2* (-1.3,-1.1)

APC, the annual percentage of change; AAPC, the average annual percent change; CI, confidence interval; ^*^p<0.001, ^**^p<0.05.

### Age–period–cohort analysis

#### Age effect

**Figure 5:**
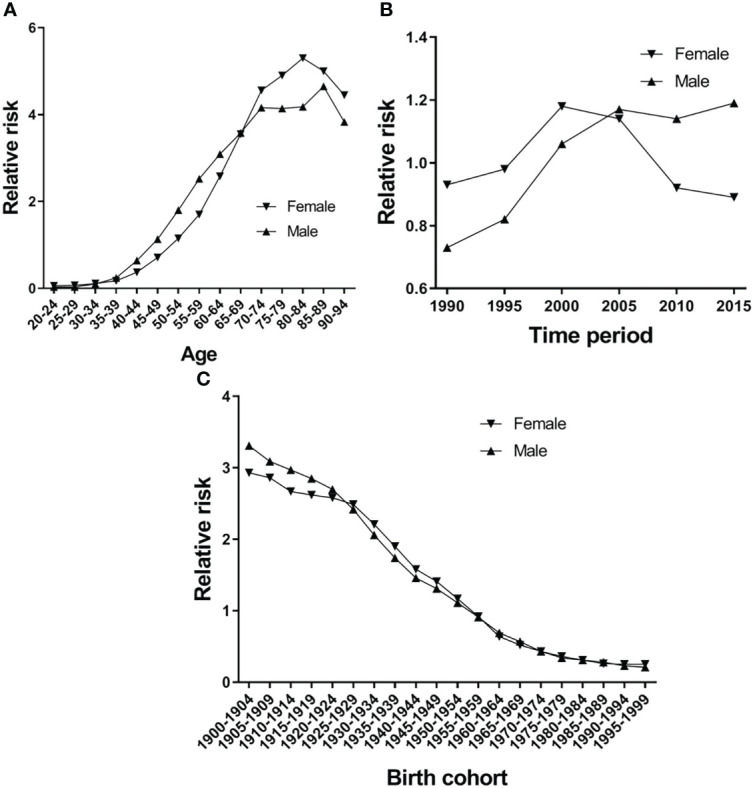
Esophageal cancer incidence relative risk due to **(A)** age; **(B)** period; and **(C)** cohort by using age-period-cohort model analysis with the intrinsic estimator period.

**Figure 6:**
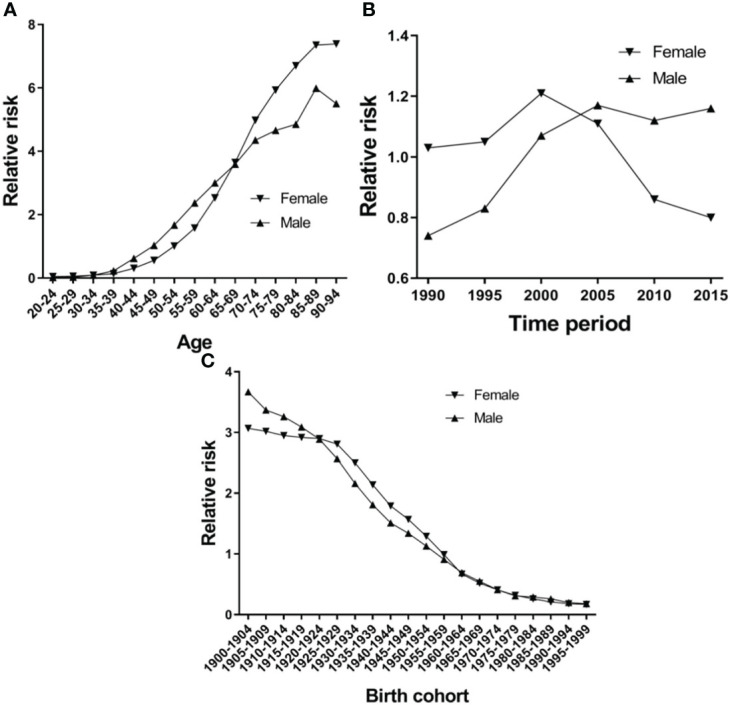
Esophageal cancer mortality relative risk due to **(A)** age; **(B)** period; and **(C)** birth by using age-period-birth model analysis with the intrinsic estimator period.

**Figure 5A** shows the EC RR of incidence by gender. The RR of EC increased with age in both females and males, except for the 80-84-year-old age group in females and the 85–89-year-old age group in males, which began to show a reduction. Females aged 50–94 and males aged 45–94 are two risk groups with an RR > 1 in incidence (**Table 3**). **Figure 6A** shows the EC RR of mortality by gender. The risk of EC increased with age in females and males, except for the 85–89-year-old male age group. Females aged 50–94 and males aged 45–94 are two risk groups with an RR > 1 in mortality (**Table 4**).

**Table 3:** Age–period–cohort (APC) model analysis results of esophageal cancer incidence in China.

Variables	Female		Male	
Case	Coef,95%CI	RR	Coef,95%CI	RR
**Age**				
20-24	-2.82* (-2.86,-2.79)	0.06	-3.62* (-3.66,-3.59)	0.03
25-29	-2.63* (-2.65,-2.6)	0.07	-3.39* (-3.42,-3.37)	0.03
30-34	-2.2* (-2.22,-2.17)	0.11	-2.32* (-2.33,-2.3)	0.1
35-39	-1.78* (-1.8,-1.76)	0.17	-1.41* (-1.42,-1.4)	0.24
40-44	-0.99* (-1.01,-0.98)	0.37	-0.45* (-0.46,-0.44)	0.64
45-49	-0.34* (-0.36,-0.33)	0.71	0.12* (0.11,0.13)	1.13
50-54	0.14* (0.13,0.15)	1.15	0.59* (0.58,0.59)	1.8
55-59	0.53* (0.52,0.54)	1.7	0.92* (0.92,0.93)	2.52
60-64	0.95* (0.94,0.95)	2.58	1.13* (1.12,1.13)	3.09
65-69	1.27* (1.26,1.27)	3.55	1.28* (1.27,1.28)	3.58
70-74	1.52* (1.51,1.52)	4.56	1.43* (1.42,1.43)	4.16
75-79	1.59* (1.58,1.59)	4.9	1.42* (1.42,1.43)	4.14
80-84	1.67* (1.66,1.68)	5.3	1.43* (1.42,1.44)	4.18
85-89	1.61* (1.6,1.62)	5	1.54* (1.53,1.54)	4.65
90-94	1.49* (1.48,1.51)	4.45	1.34* (1.33,1.36)	3.83
**Period**				
1990	-0.07* (-0.08,-0.07)	0.93	-0.32* (-0.32,-0.31)	0.73
1995	-0.02* (-0.02,-0.02)	0.98	-0.2* (-0.21,-0.2)	0.82
2000	0.17* (0.17,0.17)	1.18	0.06* (0.06,0.06)	1.06
2005	0.13* (0.13,0.13)	1.14	0.16* (0.16,0.16)	1.17
2010	-0.08* (-0.09,-0.08)	0.92	0.13* (0.13,0.13)	1.14
2015	-0.12* (-0.13,-0.12)	0.89	0.17* (0.17,0.18)	1.19
**Cohort**				
1900-1904	1.07* (1.03,1.11)	2.93	1.2* (1.14,1.26)	3.31
1905-1909	1.05* (1.03,1.07)	2.86	1.13* (1.11,1.15)	3.09
1910-1914	0.98* (0.97,1)	2.67	1.09* (1.07,1.1)	2.97
1915-1919	0.96* (0.95,0.98)	2.62	1.05* (1.04,1.06)	2.85
1920-1924	0.95* (0.94,0.96)	2.58	0.99* (0.98,1)	2.7
1925-1929	0.91* (0.9,0.92)	2.49	0.88* (0.88,0.89)	2.42
1930-1934	0.79* (0.78,0.8)	2.21	0.72* (0.71,0.73)	2.06
1935-1939	0.64* (0.63,0.65)	1.9	0.56* (0.55,0.56)	1.74
1940-1944	0.46* (0.45,0.47)	1.58	0.38* (0.37,0.39)	1.46
1945-1949	0.34* (0.33,0.35)	1.41	0.27* (0.26,0.28)	1.31
1950-1954	0.16* (0.15,0.17)	1.17	0.11* (0.1,0.12)	1.11
1955-1959	-0.08* (-0.1,-0.07)	0.92	-0.09* (-0.1,-0.08)	0.91
1960-1964	-0.44* (-0.45,-0.42)	0.64	-0.37* (-0.38,-0.35)	0.69
1965-1969	-0.66* (-0.67,-0.64)	0.52	-0.57* (-0.58,-0.56)	0.57
1970-1974	-0.85* (-0.86,-0.83)	0.43	-0.84* (-0.86,-0.83)	0.43
1975-1979	-1.01* (-1.04,-0.99)	0.36	-1.09* (-1.11,-1.07)	0.34
1980-1984	-1.16* (-1.2,-1.13)	0.31	-1.16* (-1.18,-1.13)	0.31
1985-1989	-1.34* (-1.39,-1.3)	0.26	-1.26* (-1.29,-1.23)	0.28
1990-1994	-1.38* (-1.44,-1.32)	0.25	-1.46* (-1.51,-1.41)	0.23
1995-1999	-1.4* (-1.52,-1.28)	0.25	-1.54* (-1.64,-1.44)	0.21
AIC	91.7		69.52	
BIC	6952.55		4889.83	
Deviance	7186.54		5123.82	

*p<0.05. BIC, Bayesian information criterion; AIC, Akaike’s information criterion.

**Table 4:** Age–period–cohort (APC) model analysis results of esophageal cancer mortality in China.

Variables	Female		Male	
Case	Coef,95%CI	RR	Coef,95%CI	RR
**Age**				
20-24	-3.07* (-3.12,-3.02)	0.05	-3.88* (-3.92,-3.84)	0.02
25-29	-2.84* (-2.88,-2.8)	0.06	-3.59* (-3.62,-3.56)	0.03
30-34	-2.36* (-2.39,-2.33)	0.09	-2.43* (-2.45,-2.41)	0.09
35-39	-1.96* (-1.99,-1.94)	0.14	-1.49* (-1.5,-1.48)	0.23
40-44	-1.16* (-1.18,-1.14)	0.31	-0.48* (-0.49,-0.47)	0.62
45-49	-0.57* (-0.59,-0.56)	0.56	0.03* (0.02,0.04)	1.03
50-54	0.01 (0,0.02)	1.01	0.51* (0.51,0.52)	1.67
55-59	0.46* (0.45,0.46)	1.58	0.86* (0.86,0.87)	2.37
60-64	0.93* (0.92,0.93)	2.53	1.1* (1.09,1.1)	3
65-69	1.29* (1.29,1.3)	3.65	1.28* (1.27,1.28)	3.59
70-74	1.6* (1.6,1.61)	4.98	1.47* (1.47,1.48)	4.35
75-79	1.78* (1.77,1.79)	5.93	1.54* (1.53,1.54)	4.66
80-84	1.9* (1.89,1.91)	6.7	1.58* (1.57,1.59)	4.85
85-89	2* (1.98,2.01)	7.35	1.79* (1.78,1.8)	5.99
90-94	2* (1.98,2.01)	7.39	1.71* (1.69,1.72)	5.5
**Period**				
1990	0.03* (0.02,0.04)	1.03	-0.3* (-0.31,-0.3)	0.74
1995	0.05* (0.04,0.05)	1.05	-0.19* (-0.19,-0.19)	0.83
2000	0.19* (0.19,0.2)	1.21	0.07* (0.07,0.07)	1.07
2005	0.11* (0.1,0.11)	1.11	0.16* (0.15,0.16)	1.17
2010	-0.15* (-0.16,-0.15)	0.86	0.12* (0.11,0.12)	1.12
2015	-0.23* (-0.23,-0.22)	0.8	0.15* (0.15,0.15)	1.16
**Cohort**				
1900-1904	1.12* (1.09,1.16)	3.07	1.3* (1.25,1.35)	3.67
1905-1909	1.11* (1.09,1.13)	3.02	1.22* (1.2,1.23)	3.37
1910-1914	1.08* (1.07,1.1)	2.95	1.18* (1.17,1.2)	3.26
1915-1919	1.07* (1.06,1.08)	2.92	1.13* (1.12,1.14)	3.09
1920-1924	1.07* (1.05,1.08)	2.9	1.06* (1.05,1.07)	2.89
1925-1929	1.03* (1.02,1.05)	2.81	0.94* (0.94,0.95)	2.57
1930-1934	0.92* (0.91,0.93)	2.5	0.77* (0.76,0.78)	2.16
1935-1939	0.76* (0.75,0.77)	2.14	0.59* (0.58,0.6)	1.81
1940-1944	0.58* (0.57,0.59)	1.79	0.41* (0.4,0.42)	1.51
1945-1949	0.45* (0.44,0.47)	1.57	0.29* (0.28,0.3)	1.34
1950-1954	0.26* (0.24,0.27)	1.29	0.12* (0.11,0.13)	1.13
1955-1959	-0.01 (-0.03,0.01)	0.99	-0.09* (-0.1,-0.08)	0.91
1960-1964	-0.39* (-0.42,-0.37)	0.67	-0.38* (-0.39,-0.36)	0.69
1965-1969	-0.66* (-0.68,-0.63)	0.52	-0.6* (-0.61,-0.58)	0.55
1970-1974	-0.9* (-0.92,-0.87)	0.41	-0.9* (-0.91,-0.88)	0.41
1975-1979	-1.13* (-1.16,-1.09)	0.32	-1.16* (-1.18,-1.14)	0.31
1980-1984	-1.33* (-1.38,-1.28)	0.26	-1.24* (-1.27,-1.22)	0.29
1985-1989	-1.58* (-1.65,-1.52)	0.21	-1.37* (-1.4,-1.33)	0.26
1990-1994	-1.69* (-1.78,-1.6)	0.18	-1.59* (-1.66,-1.53)	0.2
1995-1999	-1.77* (-1.96,-1.58)	0.17	-1.7* (-1.83,-1.57)	0.18
AIC	69.8		67.37	
BIC	5000.48		4700.62	
Deviance	5234.47		4934.61	

*p<0.05. BIC, Bayesian information criterion; AIC, Akaike’s information criterion.

#### Period effect

**Figure 5B** presents the RR of incidence, which rose in time period groups 1990 and 2000, decreased in time period groups 2000, 2005, 2010 and 2015, and group 2015 was lower than group 1990 in females. There is a continuing trend of growth in males, except for groups 2010 and 2015, which were higher than group 1990 (**Figure 5B**). Period groups 2000 and 2005 are two risk groups with an RR > 1 in females in incidence, but there are four time period groups (2000, 2005, 2010, and 2015) for males (**Table 3**). The time period effect pattern of RR was similar between incidence and mortality (**Figure 6A**). Period groups 1990, 1995, 2000, and 2005 are four risk groups with an RR > 1 in females regarding mortality, but 2000, 2005, 2010, and 2015 are another four groups with an RR > 1 in males (**Table 4**).

#### Cohort Effect

**Figures 5C** and **6C** show the birth cohort RR of incidence and mortality in both sexes, respectively. Overall, both curves show a downward trend in incidence and mortality. In males, the 1900–1959 birth cohort showed a faster decline in incidence (**Figure 5C**) and the 1900–1964 birth cohort in mortality (**Figure 6C**), and then it tends to be stable. The 1900–1954 birth cohort is a risk group with an RR > 1 in incidence and mortality in both sexes (**Tables 3** and **4**).

## Discussion

The global ASIR and ASMR of esophageal cancer have decreased over the past three decades, and the numbers of new EC cases and deaths and disability-adjusted life years have increased as a result of population growth and aging (2). Approximately 70% of cases occur in men, and there is a two-to-three-fold difference in incidence and mortality rates between the sexes (3). Since at least the early 1970s, vast areas of Asia have been known to have high rates of esophageal cancer extending from China and Mongolia to the Caspian Sea (2, 15). More than 90% of EC cases in this part of the world are of the esophageal squamous cell carcinoma type (16, 17). Case-cohort and control studies have found that consumption of hot tea, opium use, low intake of fresh fruits and vegetables, exposure to polycyclic aromatic hydrocarbons, indoor air pollution, low socio-economic status, and lack of access to piped water are also associated with a higher risk of esophageal squamous cell carcinoma (18–20). Age, gender, and area were independent risk factors for EC incidence (5). However, few studies have examined the trends in age-period-cohort incidence and mortality rates of EC.

This study presented the trends in incidence and mortality of EC in China. The trends of the ASIR and ASMR of EC showed a slight decrease from 1990 to 1997. However, this trend reversed in 1998 and peaked in 2004, which may have been driven by rapid economic transitions, urbanization, and political reform. Based on previous studies, we can say that China’s urbanization process has been developing faster than its economic growth since 2004 (19, 21). Besides, since 2000, a national screening programme has become available at 17 sites in Hebei Province (22). The “Four Frees and One Care” policy was announced by the Chinese government in 2003 (23). Slight increases were observed in EC ASMR from 1999 to 2004, which might be mainly associated with the early diagnosis and early treatment of esophageal cancer in rural areas. After that, both ASIR and ASMR decreased, which may be attributed to improved medical care. The joinpoint regression analysis showed that there are similar trends in the ASIR and ASMR in both sexes (six trends in females, four trends in males; **Tables 1** and 2), although overall AAPC values < 0 for both genders, what we need to pay attention to is the APC > 0 from 2016 to 2019 and its indication of an upward trend. This situation requires effective measures to reduce the increasing incidence and mortality rate of EC in China.

Age, period, and cohort effects affect the risks of disease incidence and mortality in specific ways, and this analysis can provide information about the underlying causes of cancer incidence and death (14). There have been several studies using APCM to analyze the incidence or mortality of GBD diseases (5–8, 10, 14). Our study found that the RR remarkably increased with advancing age; specifically, the RR began to increase in the 35–39 age group, continued to rise until age 80–84 in females and 85–89 in males, when it began to decline. The cancer burden among adults 85 years and older is relatively unknown (24). In our opinion, 85 and older have not received sufficient attention. In the treatment of older patients, there are numerous comorbidities, functional declines, cognitive impairment, and undertreatment. Besides, we did not include the 95 + age group, so we cannot predict how the curve continues. All of these factors may be related to China’s aging transition and contribute to the increasing age effect in EC incidence and mortality in older people in China. Period effects are often influenced by a complicated set of environmental factors and historical events, such as epidemics of infectious diseases and socio-economic development (25). Drinking alcohol and smoking tobacco are considered to be important risk factors for EC (26). The RR trend of time period between males and females is different in China, smoking tobacco and alcohol drinking might be associated with the inconsistent results. In this study, females did not seem to be affected by period effects, and the RR continued to rise in incidence and mortality in males by period. Cohort effects in EC incidence and mortality showed continuously decreasing trends. The birth cohort showed a faster decline in incidence and mortality in males, after which it tends to be stable in both sexes. One reason for this was that more of the later cohorts had an improved awareness of health and disease prevention and had received a good education compared to the earlier cohorts (27).

There are some limitations to this study. First, the APCM is a descriptive analysis that use a community as a unit of observation and analysis, which could lead to ecological fallacies, the results are not necessarily valid for individuals. Second, the APCM of estimated parameters can only provide evidence for etiology studies. Third, the study could not estimate trends for the incidence and mortality rate in both rural and urban China, owing to insufficient data. The epidemiology of EC in rural and urban areas needs to be analyzed in the future.

## Conclusion

The overall incidence and mortality of esophageal cancer in China shows an increased and then decreased trend from 1990 to 2019, and the AAPC was decreased in incidence and mortality from 1990 to 2019. The RR of incidence and mortality of esophageal cancer increased with age and time period and decreased with birth cohort. Therefore, more effective measures need to be taken to enhance the protection of the elderly, who are at particularly high risk.

## Data availability statement

Publicly available datasets were analyzed in this study. This data can be found here: http://ghdx.healthdata.org/gbd-results-tool.

## Author contributions

FL wrote the manuscript. ZW and JZ conceived the study and provided guidance. HSL and XS collected and analyzed data. All authors contributed to the article and approved the submitted version.

## Acknowledgments

We thank International Science Editing (http://www.internationalscienceediting.com) for editing this manuscript.

## Conflict of interest

The authors declare that the research was conducted in the absence of any commercial or financial relationships that could be construed as a potential conflict of interest.

## Publisher’s note

All claims expressed in this article are solely those of the authors and do not necessarily represent those of their affiliated organizations, or those of the publisher, the editors and the reviewers. Any product that may be evaluated in this article, or claim that may be made by its manufacturer, is not guaranteed or endorsed by the publisher.
