# Supplementary material for: Trends in incidence and mortality of esophageal cancer in China 1990−2019: A joinpoint and age-period-cohort analysis
Source: Front Oncol. 2022 Aug 15;12:887011. doi: 10.3389/fonc.2022.887011 (PMC9420985; doi:10.3389/fonc.2022.887011)
Supplement: Supplementary file 2 [file Table_2.docx]

**TABLE S2** │ The data collection process

Visit website <https://ghdx.healthdata.org/gbd-results-tool.>


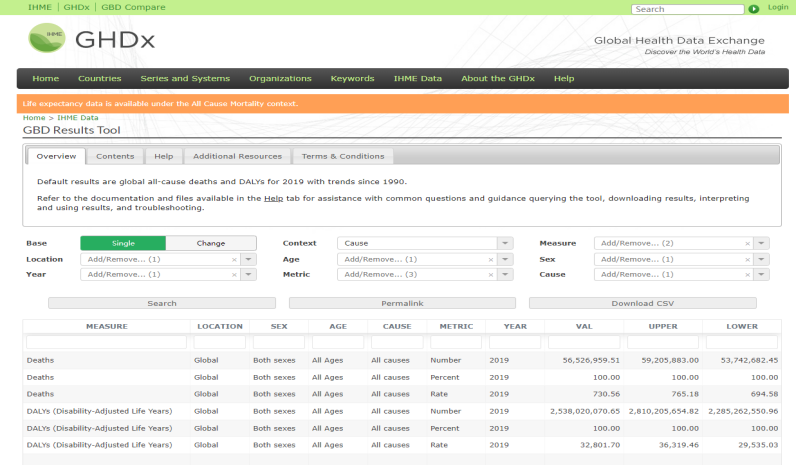


Location = China; Year = select all (from 1990 to 2019); Context = Cause; Age = age-standardized, 1-4, 5-9, 10-14, 15-19, 20-24, 25-29, 30-34, 35-39, 40-44, 45-49, 50-54, 55-59, 60-64, 65-69, 70-74, 75-79, 80-84, 85-89, 90-94; Metric = Number and Rate; Measure = Deaths, DALYs, and Incidence; Sex= Both, Female, and Male; Cause = B.1.4 Esophageal cancer.
